# Genetic diversity analysis of cultivated Korarima [*Aframomum corrorima* (Braun) P.C.M. Jansen] populations from southwestern Ethiopia using inter simple sequence repeats (ISSR) marker

**DOI:** 10.1186/s40709-017-0073-z

**Published:** 2018-01-08

**Authors:** Dagmawit Chombe, Endashaw Bekele

**Affiliations:** Department of Microbial Cellular, and Molecular Biology, College of Computational and Natural Sciences, Addis Ababa University, P.O. Box: 1176, Addis Ababa, Ethiopia

**Keywords:** *Aframomum corrorima*, Korarima, Inter simple sequence repeats (ISSR), Genetic diversity, Conservation

## Abstract

**Background:**

Korarima (*Aframomum corrorima*) is a perennial and aromatic herb native and widely distributed in southwestern Ethiopia. It is known for its fine flavor as a spice in various Ethiopian traditional dishes. Few molecular studies have been performed on this species so far. In the present paper, the ISSR technique was employed to study the genetic diversity in populations of cultivated *A. corrorima*.

**Results:**

Seven ISSR primers produced a total of 86 clearly scorable DNA bands. High levels of genetic diversity were detected in cultivated *A. corrorima* (percentage of polymorphic bands = 97.67%, gene diversity = 0.35, Shannon’s information index = 0.52). Analysis of molecular variance (AMOVA) showed that 27.47% of the variation is attributed to the variation among populations and 72.53% to the variation within populations. The F_st_ (0.28) value showed a significant (*p* < 0.0001) genetic differentiation among populations. This was supported by the high coefficient of gene differentiation (G_st_ = 0.32) and low estimated gene flow (Nm = 1.08). A neighbor-joining dendrogram showed that the thirteen cultivated populations were separated into three clusters, which was in good accordance with the results provided by the two dimensional and three dimensional coordinate analyses. However, the clusters did not reveal clear pattern of populations clustering according to their geographic origin. This could be due to human mediated transfer of genetic material among different localities.

**Conclusion:**

The genetic diversity in populations of *A. corrorima* from the southwestern part of Ethiopia was relatively high. This finding should be taken into account when conservation actions, management policies for the species and site identification for in situ and ex situ conservation strategies are developed. Mizan Teferi II population displayed the highest genetic diversity; this population should be considered as the key site in designing conservation strategies for this crop. In addition, Jimma I and Jimma II populations with lowest genetic diversity, should also be considered due to the putative risk of extinction that they face because of the low genetic diversity.

## Background

Korarima [*Aframomum corrorima* (Braun) P.C.M. Jansen] belongs to the family Zingiberaceae and it is native to Ethiopia. It is a perennial tropical aromatic herb, often of large size, bearing flowers either terminally on aerial leaf shoots or from ground level [1]. Korarima grows usually with strong fibrous subterranean scaly rhizomes and with leafy stems reaching 1–2 m high. The position of stigma in the flower is below or against the base of the thecae of the anther. It is usually self-pollinated. Occasionally, cross-pollination by insects is possible due to the presence of large nectaries at the top of the ovaries [2].

*Aframomum corrorima* is widely distributed in Ethiopia, Sudan (southwestern, Aloma Plateau), Uganda (western), Tanzania (Usambara Mountains) and Eritrea [1]. The use of korarima is known widely in Ethiopia, from Keffa, Gamu Gofa, Sidama, Illubabor, Bale, Welega to East and West Gojam. Cultivation of the plant has been reported not only in places where it grows wild, but also in the Lake Tana and Gelemso areas (Harerge) [3]. Korarima can be propagated either by seed or by cutting of its clumps, though the latter is by far the most common method, as it yields earlier and ensures a true-to-type propagation than the former [4]. Because of the predominant vegetative propagation method employed, the maximum number of korarima landraces identified in three administrative zones studied by Eyob et al. [2] was three.

Korarima is a spice and medicinal plant of economic importance and an indigenous and endangered species of Ethiopia [5]. The seeds of korarima are mainly used as spices in traditional Ethiopian dishes [6]. It is a source of income for growers as its seeds reach high prices in local and export markets. Korarima parts are used in traditional medicine for humans and cattle. Also korarima is an important plant for soil conservation as the rhizomes and leaves spread on the ground covering and protecting the soil from erosion in hilly areas all year round [7]. Compared with other *Aframomum* species, the seeds of korarima have less pungent, milder and sweeter flavor. There is a demand for korarima in the neighboring countries and in Saudi Arabia where it has long been highly prized as a spice while there is a potential to expand its use to other regions [8]. This spice could be developed into an important commercial product if necessary attention is given to its research and genetic improvement.

The moist montane forested land of the southwestern part of Ethiopia is dominated by forests that harbor genetic resources of vast social, economic and environmental importance. Every household has its own forest land with big trees used to hang traditional bee hives, to provide shade for different herb plants, like korarima and coffee, and to supply fruits, firewood and timber. This cultural practice helps the farmers to conserve the natural forest [9]. However, the forests are mostly fragmented mainly due to clear-cutting forests for expansion of agricultural fields which are currently being used for cereal production such as teff, maize, wheat, barley and perennial crops like *Enset ventricosum.* Expansion of settlements, both urban and rural, and cultivation are creating “Vegetation Islands” [10].

Korarima is one of the species in which genetic erosion is a real threat since its natural habitat, the humid mountain forests of southwestern Ethiopia, is being decimated at an increasing rate [9]. To minimize the loss of korarima genetic resources, collecting germplasms from different geographic locations and conserving them at in situ and ex situ conservation sites is the best strategy for conservation and sustainable utilization. However, to design the appropriate conservation strategy, analyzing the genetic diversity is needed. Thus, the aim of this study was to evaluate the genetic diversity in cultivated *A. corrorima* from southwestern Ethiopia, by using inter simple sequence repeat (ISSR) markers, to gain knowledge regarding conservation issues and best use of genetic resources.

## Methods

### Plant material

**Fig. 1:**
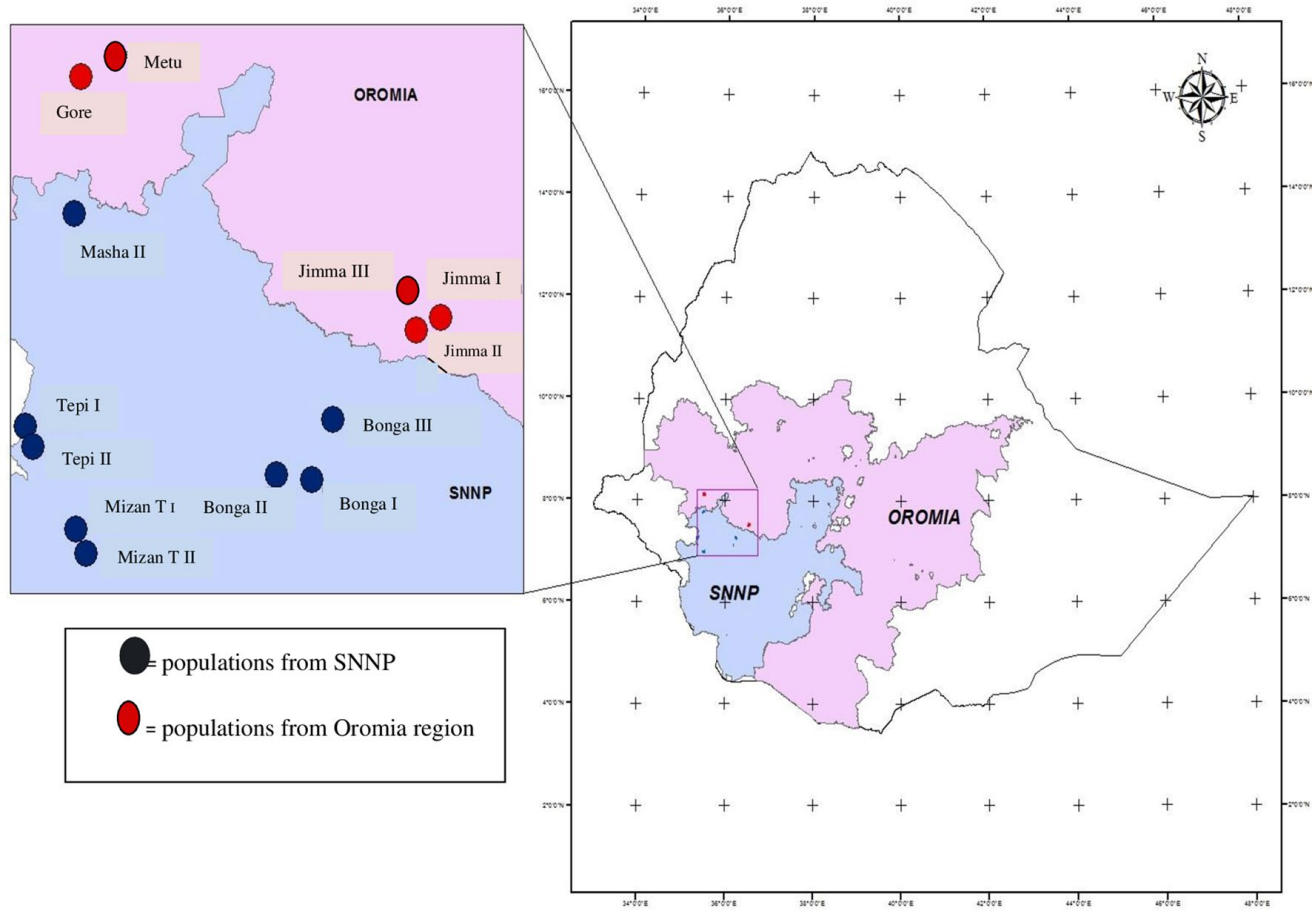
Regional map of Ethiopia showing the cultivated korarima collection sites (*SNNPR* Southern Nations, Nationalities, and People’s Region). The map was constructed based on geographic coordinates and elevation data gathered from each collection sites using global positioning system (GPS)

One hundred twenty nine (129) individuals, which corresponded to 13 populations (Table 1), were sampled across five geographic zones (Illubabor, Jimma, Sheka, Kefa and Bench Maji) in southwestern part of Ethiopia (Fig. 1). Letters of consent were obtained from all concerned authorities. The average kororima production area among the farmer’s field considered was between 0.05 and 0.15 ha, which is rather a small area. Therefore, only nine to ten individuals of *A. corrorima* were randomly chosen from each of the 13 cultivated populations. The distance between sampled plants within each population was at least 20 m, in order to increase the likelihood of sampling inter-individual variation. Two very young healthy leaves were collected from each plant and sealed within plastic bags containing Silica gel. The samples were taken to the laboratory and stored at room temperature until used for DNA extraction.

**Table 1:** Description of *A. corrorima* samples collected from different sites in Ethiopia

Zone	Specific location	Population name (code)	Samples	Latitude	Longitude	Altitude (masl)
Illubabor Zone	Sege-Tageta	Gore (GC1-10)	10	7°01′23.4″N	36°32′14.9″E	1924
	Adele Bise	Metu (MC1-10)	10	6°57′38.9″N	36°49′15.9″E	1655
Jimma Zone	Sebeka Dibiya 1	Jimma I (JC1-10)	10	7°31′18.9″N	36°32′03.9″E	1956
	Sebeka Dibiya 3	Jimma II (jC1-10)	10	7°31′50.6″N	36°33′02.6″E	2045
	Sebeka Dibiya 2	Jimma III (JW1-10)	10	7°31′50.6″N	36°33′02.6″E	2045
Bench Magi Zone	Fanika-1	Mizan Teferi I (mtc1-10)	9	7°00′51.2″N	35°26′03.2″E	1336
	Fanika-2	Mizan Teferi II (MTC1-10)	10	7°00′52.5″N	35°25′59.6″E	1348
Sheka Zone	Beta	Masha (MaC1-10)	10	7°45′37.2″N	35°29′04.1″E	2172
	Kubito-1	Tepi I (TC1-10)	10	7°18′58.3″N	35°23′00.5″E	1885
	Kubito-2	Tepi II (tc1-10)	10	7°19′09.4″N	35°22′36.4″E	1904
Kefa Zone	01 kebele	Bonga I (BC1-10)	10	7°17′18.4″N	36°13′30.3″E	1657
	Beha	Bonga II (BoC1-10)	10	7°15′42.1″N	36°14′44.4″E	1701
	Around college	Bonga III (bC1-10)	10	7°10′11.7″N	36°13′21.1″E	1847

Zone is a collection of Woredas together; the GPS system used was universal transverse mercator coordinates (UTM)

*masl* meter above sea level

### DNA extraction

Total genomic DNA was isolated from about 1 g of pulverized leaf sample following a modified CTAB method employing triple extractions to yield optimal amounts of DNA [11]. Genomic DNA was loaded on an agarose gel (Sigma, Steinheim, Germany) of 0.83% and electrophoresed at a constant voltage of 80 V for about 45 min. Then, the gel was stained with ethidium bromide. To check the quality of sample DNA, a gel picture was taken under UV trans-illuminator by Biodoc Analyse 2.0 with digital Cannon camera (Biometra, Gottingen, Germany). Among the three extractions, the one with the best quality and quantity was screened from the gel picture and used for subsequent PCR analysis.

### PCR amplification and gel electrophoresis

The polymerase chain reaction (PCR) was conducted by using a Biometra 2000 T3 Thermo cycler (Biometra, Gottingen, Germany). PCR amplification was carried out in a 25 μl reaction as follows: template DNA (10 ng μl^−1^), dNTPs (0.3 mM), Taq buffer (1× Thermopol reaction buffer) (Thermo Fisher Scientific, Massachusetts, USA), MgCl_2_ (1.5 mM), primer (0.3 μM) and Taq polymerase (1 U μl^−1^) and sterile double-distilled water added to 25 μl. The amplification program was 4 min pre-heating and initial denaturation at 94 °C, followed by 40 cycles of 15 s at 94 °C, 1 min at the specific annealing temperature for each primer, and 1 min and 30 s extension at 72 °C. The lid temperature was held at 105 °C. A final extension for 7 min at 72 °C followed. The PCR products were stored at 4 °C until loaded on gel for electrophoresis.

The amplified products were separated by electrophoresis using 1.67% agarose gels in 1 × TBE. A 100 bp DNA ladder (Thermo Fisher Scientific, Massachusetts, USA) was used to estimate the size of each PCR product. Electrophoresis was carried out for 2 h at constant voltage of 100 V. The gels were stained with ethidium bromide and observed on a UV light and photographed using Biodoc Analyse 2.0 gel documentation system (Biometra, Gottingen, Germany).

### ISSR data analysis

Since ISSR markers are dominant and *Aframomum corrorima* is a diploid with chromosome number (2n = 48) [12], it is assumed that each band represents the phenotype at a single biallelic locus. Unambiguously reproducible amplified fragments were scored manually for presence (1) or absence (0) after photographing the gel with the Biodoc Analyse. Strong, reproducible and clearly distinguishable bands were used in the analysis. The reproducibility of the amplification pattern was checked by running repeated PCR amplifications for randomly chosen individual *A. corrorima* samples using selected primers. The resulting binary data matrix of the ISSR phenotypes was analyzed using POPGENE version 1.31 [13], to estimate the following genetic diversity parameters: the percentage of polymorphic bands (PPB), genetic diversity index (GD) [14], Shannon’s information index (I), gene differentiation coefficient (G_ST_) and the level of gene flow (N_m_) from G_st_ according to [15] where N_m_ = 0.5 (1 − G_st_)/G_st_). Nei’s unbiased genetic identity (I) [16] and genetic distance (D) between populations were also computed using this software.

The hierarchical analysis of molecular variance (AMOVA) was used to calculate variance components within and among populations. Partitioning the total variation to different hierarchical level was done using Arlequin (version 3.0b) software [17]. The un-weighted pair group method with arithmetic mean (UPGMA) [18] was used to analyze and compare the individual genotypes as well as populations and generates phenogram using NTSYS-PC (version 2.1) software [19]. The neighbor-joining (NJ) method [20, 21] was used to compare individual genotypes and evaluate patterns of genotype clustering using Free Tree 0.9.1.50 software [22].

The pattern of variation among individual samples was further examined by principal coordinate analysis (PCoA) which was performed based on Jaccard’s coefficient of similarity [23]. The calculation of Jaccard’s coefficient of similarity was made with PAST (version 1.18) software [24]. The first three axes were later used to plot two and three dimensional coordinate analysis with STATISTICA (version 6) software [24].

## Results

**Fig. 2:**
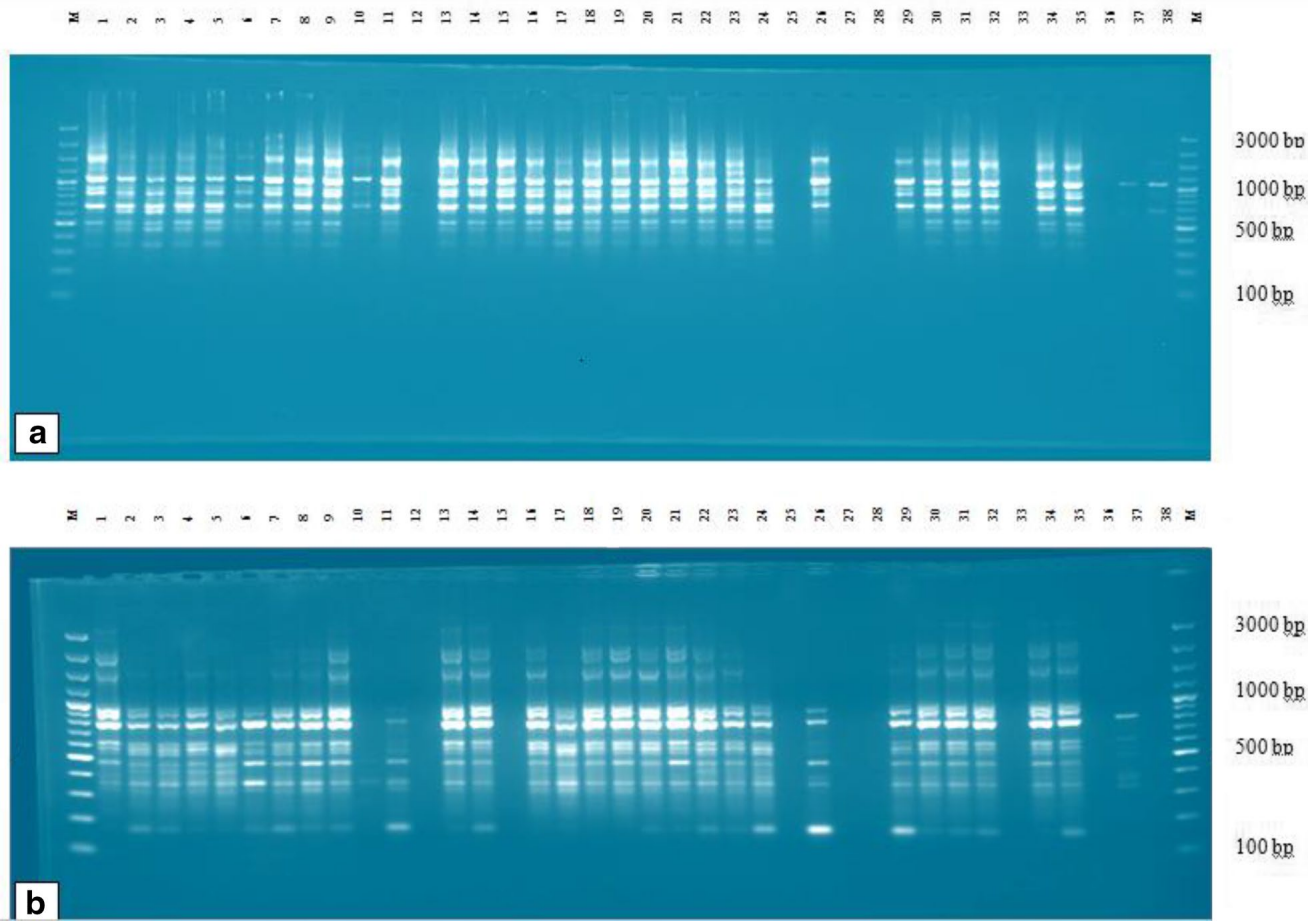
ISSR profile from amplification of genomic DNA of 38 individuals of *A. corrorima* with primers UBC-825 (**a**) and UBC-834 (**b**). (M = 100-bp ladder)

Out of the 18 primers tested initially, seven primers gave clear banding pattern and were selected and used in this study (Table 2). The molecular weight of the bands amplified using the primers was in the range of 100–3000 bp (Fig. 2). A total of 86 reproducible bands were scored from 129 *A. corrorima* individuals and 79 (91.86%) were found to be polymorphic. The total number of bands per primer varied from 6 (UBC-853) to 18 (UBC-841), with an average of 12.29 fragments per primer. The highest values of Nei’s gene diversity (0.43) and Shannon’s information index (0.62) were exhibited by primer UBC-835. In contrast, primer UBC-853 showed the lowest values of Nei’s gene diversity and Shannon’s information index (0.27 and 0.42, respectively). The mean Nei’s gene diversity and Shannon’s information index values for all the primers were 0.34 and 0.51, respectively (Table 3).

**Table 2:** List of primers, their sequence, annealing temperature and amplification pattern used for PCR optimization

	Primer	Primer sequences	Annealing temperature (°C)	Amplification pattern
1	UBC-810	(CA)_8_T	45	No band
2	*UBC-811*	*(CA)*_*8*_ *RC*	*48*	*Very good*
3	UBC-813	(GATA)_8_	45	Fair
4	UBC-814	(AT)_8_YC	45	No band
5	UBC-815	(GA)_8_YT	48	Fair
6	UBC-817	(GT)_8_YA	45	Fair
7	UBC-822	(GT)_8_YC	45	No band
8	*UBC-825*	*(AT)*_*8*_ *T*	*45*	*Very good*
9	UBC-831	(AT)_8_YA	48	No band
10	*UBC-834*	*(AG)*_*8*_ *YT*	*45*	*Excellent*
11	*UBC-835*	*(AG)*_*8*_ *YC*	*48*	*Excellent*
12	*UBC-841*	*(GA)*_*8*_ *YC*	*48*	*Excellent*
13	UBC-843	(CT)_8_RA	45	Fair
14	UBC-846	(CA)_8_RT	48	No band
15	UBC-849	(GT)_8_YA	45	No band
16	UBC-850	(GT)_8_YC	48	Fair
17	*UBC-853*	*(TC)*_*8*_ *RT*	*48*	*Good*
18	*UBC-857*	*(AC)*_*8*_ *YG*	*45*	*Very good*

ISSR primers (UBC 1–18) were from the University of British Columbia, Canada. The seven primers used in the present study for PCR amplification are highlighted in italics

**Table 3:** Genetic diversity parameters of 7 ISSR primers selected for use in this study

Primer	NSB	NPL	PP (%)	GD	I	Size range of the bands (bp)
UBC-811	11	6	85.71	0.32	0.47	800–1500
UBC-825	13	12	92.31	0.36	0.52	500–2000
UBC-834	15	15	100	0.38	0.55	100–2000
UBC-835	11	11	100	0.43	0.62	300–2000
UBC-341	18	18	100	0.33	0.51	100–3000
UBC-853	6	5	83.33	0.27	0.42	1000–3000
UBC-857	12	12	100	0.31	0.48	150–1500
Mean	12.29	11.29	94.48	0.34	0.51	

*NSB* number of scorable bands, *NPL* number of polymorphic loci, *PP* percent of polymorphism, *GD* genetic diversity, *I* Shannon’s information index

At the population level, the percentage of polymorphic bands (PPB) ranged from 31.40% in the Jimma I population to 81.40% in the Mizan Teferi II population. Similarly, the highest values of Shannon’s diversity index and Nei’s gene diversity for cultivated korarima population was recorded for Mizan Teferi II (GD: 0.29 and I: 0.43, respectively). Although samples of Jimma I, Jimma II and Jimma III populations were collected from geographically close sites in Jimma, populations Jimma I (PPB: 31.40%, GD: 0.13 and I: 0.19) and Jimma II (PPB: 34.88%, GD: 0.15 and I: 0.22) exhibited the lowest values in all parameters of genetic diversity estimates (Table 4). However, Jimma III population showed higher values for these parameters (PPB: 68.60%, GD: 0.27 and I: 0.39) than population Jimma I and Jimma II. The average genetic diversity index value was found to be 0.35 (Table 4).

**Table 4:** Gene diversity estimators for populations of *A. corrorima* from the southwestern part of Ethiopia based on the results of seven ISSR primers

No	Population	NPL	PPB (%)	GD	I	G_ST_	N_m_
1	Gore	59	68.60	0.28	0.40		
2	Metu	38	44.19	0.20	0.29		
3	Jimma I	27	31.40	0.13	0.19		
4	Jimma II	30	34.88	0.15	0.22		
5	Jimma III	59	68.60	0.27	0.39		
6	Mizan Teferi I	59	68.60	0.26	0.38		
7	Mizan Teferi II	70	81.40	0.29	0.43		
8	Masha	49	56.98	0.23	0.34		
9	Tepi I	56	65.12	0.25	0.36		
10	Tepi II	56	65.12	0.25	0.36		
11	Bonga I	51	59.30	0.24	0.35		
12	Bonga II	56	65.12	0.29	0.41		
13	Bonga III	44	51.16	0.20	0.29		
	Overall	84	97.67	0.35	0.52	0.32	1.08

*NPL* number of polymorphic loci, *PPB* percent polymorphic bands, *GD* genetic diversity, *I* Shannon’s index, *G*_*ST*_ estimate of genetic differentiation, *N*_*m*_ estimate of gene flow

The genetic differentiation among populations (G_st_), estimated according to [13] was 0.32. Furthermore, the level of gene flow (N_m_) was estimated to be 1.08 individuals per generation between populations.

Inter-population genetic distance ranged from 0.06 to 0.41 for the 13 populations (Table 5). Among the pairwise population comparisons made within SW Ethiopian cultivated korarima populations the highest genetic distance was observed between Jimma II and Bonga III populations (jC vs bC: 0.41) and the lowest genetic distance was observed between Bonga I and Bonga III population (BC vs bC: 0.06) (Table 5).

**Table 5:** Nei’s original measure of genetic distance in 13 populations of Ethiopian *A. corrorima*

Pop ID	GC	MTCI	MTCII	MC	JW	bC	BC	BoC	MaC	JC	TC	tC	jC
GC	****												
MTCI	0.12	****											
MTCII	0.13	0.12	****										
MC	0.22	0.18	0.25	****									
JW	0.14	0.08	0.09	0.19	****								
bC	0.12	0.15	0.19	0.18	0.15	****							
BC	0.10	0.11	0.13	0.16	0.10	*0.06*	****						
BoC	0.12	0.10	0.13	0.20	0.10	0.16	0.10	****					
MaC	0.11	0.15	0.17	0.24	0.17	0.15	0.13	0.12	****				
JC	0.24	0.26	0.27	*0.36*	0.28	0.28	0.26	0.27	0.20	****			
TC	0.22	0.21	0.16	0.33	0.20	0.28	0.24	0.19	0.17	0.11	****		
tC	0.18	0.24	0.18	0.32	0.21	0.31	0.25	0.22	0.24	0.15	0.09	****	
jC	0.27	0.29	0.34	0.20	0.32	*0.41*	0.33	0.30	0.33	0.27	0.23	0.18	****

Italic values indicate the highest and lowest genetic distance between pairs of population

*GC* Gore, *MTCI* Mizan Teferi I, *MTCII* Mizan Teferi II, *MC* Metu, *JW* Jimma III, *bC* Bonga III, *BC* Bonga I, *BoC* Bonga II, *MaC* Masha, *JC* Jimma I, *TC* Tepi I, *tC* Tepi Cul, *jC* Jimma II

Analysis of molecular variance (AMOVA) was carried out on the overall ISSR data of cultivated korarima populations (Table 6), firstly without grouping the populations and then by grouping the populations based on the region or on the zone they belong. AMOVA without grouping revealed that 72.53% of the variation is attributed to the within populations variation while the remaining variation is due to the among populations variation (27.47%). The genetic differentiation among the populations was high (F_ST_: 0.28) and found to be highly significant (*p* < 0.0001).

**Table 6:** Analysis of molecular variance (AMOVA) of *A. corrorima* populations based on seven ISSR primers

Groups	Source of variation	d.f.	Sum of squares	Variance components	% of variation	Fixation index	*p* value
(A) Without grouping the populations	AP WP Total	12 109 121	241.70 482.27 723.97	1.68 Va 4.43 Vb 6.10	27.47 72.53	F_ST_: 0.28	Va and F_ST_ = 0.00
(B) Populations grouped by region	AR APWR WR Total	1 11 109 121	10.22 231.48 482.27 723.97	− 0.19 Va 1.78 Vb 4.42 Vc 6.01	− 3.19 29.55 73.64	F_ST_: 0.26 F_SC_: 0.29 F_CT_: − 0.03	Vc and F_ST_ = 0.00 Vb and F_SC_ = 0.00 Va and F_CT_ = 0.80
(C) Populations grouped by zones of origin	AZ APWZ WZ Total	4 8 109 121	117.19 124.51 482.27 723.98	0.57 Va 1.19 Vb 4.42 Vc 6.18	9.15 19.27 71.58	F_ST_: 0.28 F_SC_: 0.21 F_CT_: 0.09	Vc and F_ST_ = 0.00 Vb and F_SC_ = 0.00 Va and F_CT_ = 0.07

(A) Without grouping the populations, (B) by grouping the populations based on regions and (C) by grouping the populations according to zones of origin

*AP* among population, *WP* within population, *AR* among region, *APWR* among population within region, *WR* within region, *AZ* among zone, *APWZ* among population within zone, *WZ* within zon, *F*_*ST*_ the variance among subpopulations relative to the total variance, *F*_*SC*_ the variance among subpopulations within groups, *F*_*CT*_ the variance among groups relative to the total variance

AMOVA results obtained by grouping the populations into regions showed no variation among Oromia and Southern Nations Nationalities People (SNNP) region, while 73.64% of the variation was within the two regions. The variation among the two regions was very low and non significant (F_CT_ < 0.01; *p* > 0.05) (Table 6). On the other hand, AMOVA results obtained by grouping the populations into zones showed 9.15% of the total genetic diversity was partitioned to the variation among the five zones, compared to 71.58% that was partitioned to the variation within the zones. The genetic differentiation is moderate but non significant (F_CT_ = 0.09; *p* > 0.05) (Table 6).

**Fig. 3:**
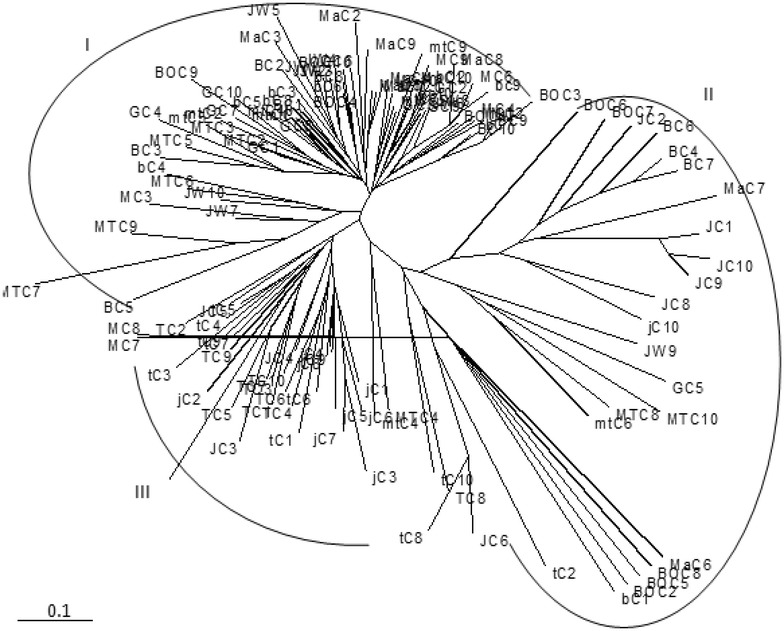
Neighbor-joining clustering of 129 individuals of *Aframomum corrorima* based on ISSR data generated from seven primers. The algorithm is based on Jaccard’s coefficients of similarity obtained after pair-wise comparison of the presence-absence fingerprint

The dendrogram derived from the neighbor-joining analysis of the whole ISSR data set (that includes 129 Ethiopian korarima genotypes), showed three major clusters (Fig. 3). Cluster I and II consist of a mixture of individuals from at least ten populations. Cluster III comprise individuals from four populations (Tepi I, Jimma II, Tepi II, Jimma I). These results show admixture of individuals in all clusters.

**Fig. 4:**
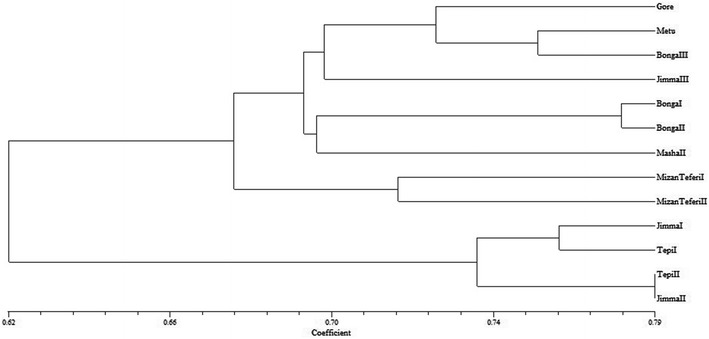
Dendrogram of the 13 populations of *Aframomum corrorima* from Ethiopia generated from ISSR data using unweighted pair group method of arithmetic means (UPGMA). The algorithm is based on Jaccard’s coefficients of similarity obtained after pairwise comparison of the presence-absence fingerprint

The dendrogram produced by UPGMA for all 13 populations is presented in Fig. 4. Two major clusters (I and II) can be observed from the dendrogram. The first major cluster (I) includes three sub clusters A, B and C. Sub-cluster A consists of four populations (Gore, Metu, Bonga III and Jimma III), sub-cluster B of three populations (Bonga I, Bonga II and Masha) and sub-cluster C of two populations (Mizan Teferi I and Mizan Teferi II). The second cluster consists of four populations (Jimma I, Jimma II, Tepi I and Tepi II) from Jimma and Tepi.

**Fig. 5:**
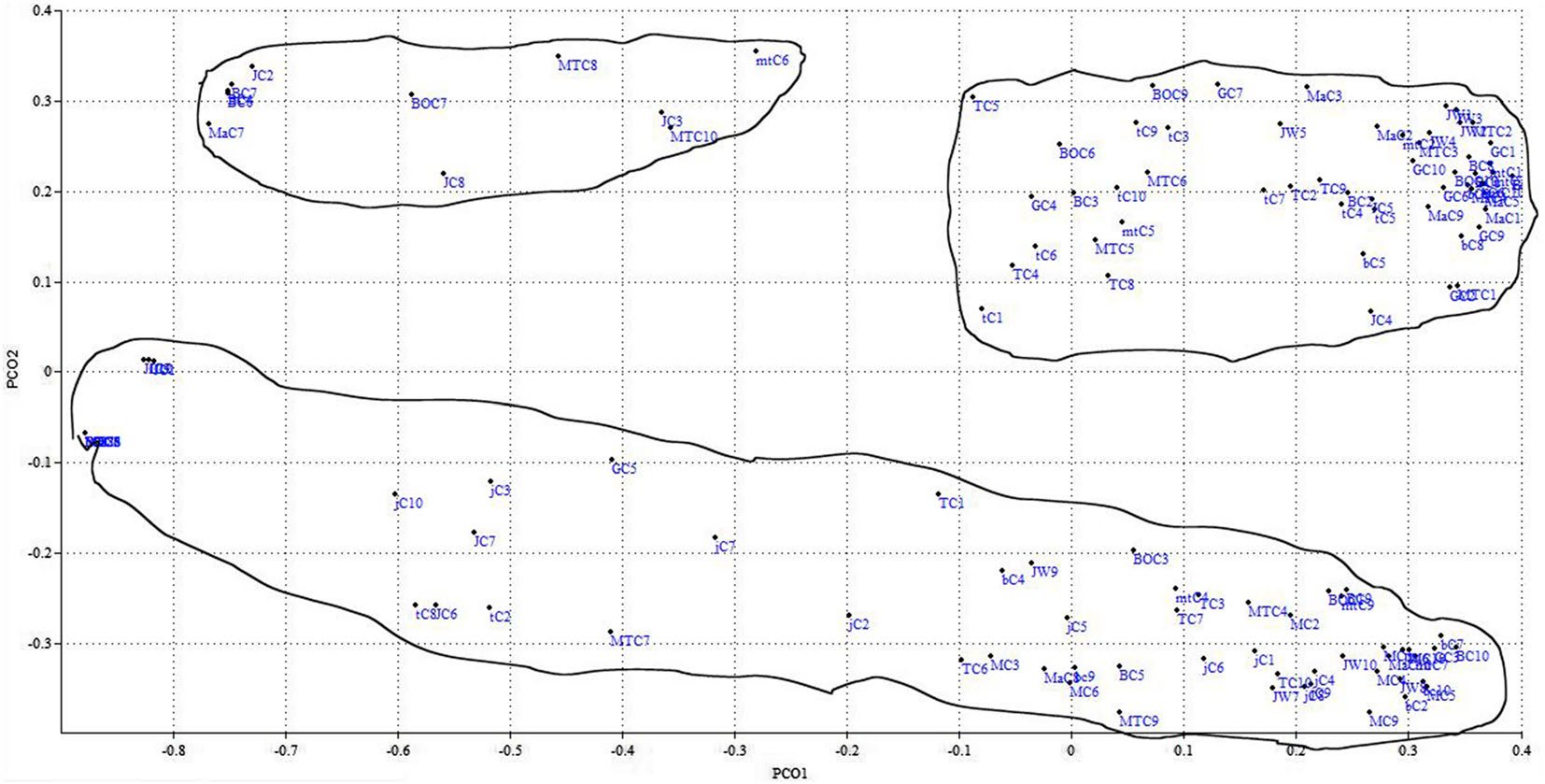
Two dimensional representation (2D) of principal coordinate analysis of genetic relationships among 129 individuals of *Aframomum corrorima* from southwestern part of Ethiopia

**Fig. 6:**
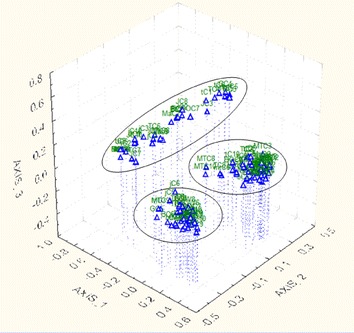
Three dimensional representation (3D) of principal coordinate analysis of genetic relationships among 129 individuals of *Aframomum corrorima* from southwestern part of Ethiopia

In the whole data-set from the seven ISSR primers, a PCoA analysis was carried out using Jaccard’s coefficient of similarity. The first three coordinates of the PCoA with Eigenvalues of 19.8, 8.1 and 5.5 (and variance of 26.4, 10.8 and 7.4%, respectively) showed the grouping of individuals using two and three dimensional coordinates. Two dimensional (2D) coordinate analyses partitioned the 129 individuals of *A. corrorima* into three clusters (Fig. 5). However, the thirteen populations failed to form separate clusters according to their respective collection origin and individuals from more than three populations were observed together in the three clusters. Similarly, in the three dimensional representation (3D) of PCoA analysis, the 129 individuals of *A. corrorima* were grouped into three clusters and they did not form any group according to their population origin (Fig. 6).

## Discussion

### Genetic diversity

The present study reports genetic diversity parameters for cultivated Ethiopian *A. corrorima* populations using ISSR marker. The extent and pattern of genetic diversity among 129 cultivated individuals of korarima from 13 populations were estimated using seven di-nucleotide ISSR markers. Based on the observed results, the application of ISSR DNA finger-printing technique was efficient and successful for revealing the diversity among individuals and populations of *A. corrorima*, scoring a range from 5 to 18 polymorphic bands. From all the populations, 86 discernible ISSR fragments were generated among which 79 were polymorphic. The overall percentage of polymorphism (94.48%) revealed by this study suggests that genetic diversity of the studied individuals and populations is high. Long-lived perennials have higher gene diversity compare to plants that are annuals or short-lived perennials [25–27]. The high diversity observed in *A. corrorima* might be due to long life span. The mean Nei’s genetic diversity (GD) and Shannon’s information index (I) values for all primers were 0.34 and 0.51 respectively, indicating an immense genetic diversity at species level. This result is in accordance with the result obtained in other perennial plants in the Zingiberaceae family like *Curcuma alismatifolia* from Thailand (GD: 0.29; I: 0.43) [28] and in the spice plant black cumin (*Nigella sativa* L.) (GD: 0.42; I: 0.54) [29] from Ethiopia, that have been recently studied with ISSR markers. The values of Nei’s genetic diversity (GD) obtained for native plants of Ethiopia like enset (GD: 0.27) [30], teff (GD: 0.27) [31] and sorgum (GD: 0.21) [32] using ISSR markers in the previous studies showed a lower value than obtained in the present study. However, this result contradicts the farm based biodiversity study of *A. corrorima* in southern Ethiopia by Eyob et al. [2]. The Shannon–Weaver and Simpson diversity indices calculated by Eyob et al. [2] indicated that the biodiversity of korarima landraces is lower when compared with other native plants of Ethiopia (enset, tef and sorghum) in all administrative zones studied [2].

The success of a crop-improvement program largely depends on the availability and knowledge of the genetic resources in a germplasm collection. Since areas of high genetic diversity mostly contribute more accessions than those of low diversity for further and future collection, breeding and conservation activities should be based in areas with high genetic diversity. Among the 13 populations from southwestern Ethiopia studied, the highest percentage of polymorphic bands (PPB), gene diversity (GD) and Shannon’s diversity index (I) respectively were recorded in Mizan Teferi II population (81.40%, 0.29 and 0.43) (Table 4). Hence this population should be the main focus for future follow up of collection and population conservation as compared to areas which possess lowest genetic diversity estimates, such as Jimma I (31.40%, 0.13 and 0.19) and Jimma II (34.38%, 0.15 and 0.22). However, these areas, too, may have rare types that one may discover in the process of evaluating for quality using a large sample size and collection. On the other hand, though Jimma III population was found in the same zone with Jimma I and Jimma II populations, the highest genetic diversity estimates were recorded in Jimma III which might be attributed to selection by local farmers. The lowest genetic diversity in Jimma I and Jimma II might be explained by the existence of uniformity possibly due to vegetative way of propagation prevailed in the area and due to single or few seed sources during its introduction to these areas.

### Genetic structure and gene flow

The overall degree of genetic differentiation of *A. corrorima,* as estimated by G_st_ (0.32) (Table 4), is much higher than the average of G_st_ = 0.19 for perennial plants and is equal to the average of G_st_ = 0.32 for dicotyledons [25, 26]. Breeding systems of plants greatly affect genetic differentiation, and selfing can result in low genetic diversity within populations [33]. In line with this, high and significant F_st_ was obtained, indicating high genetic differentiation among populations of the self pollinating *A. corrorima.* AMOVA results revealed that, of the total variation, 23.9% was attributed to among-populations differences and 76.1% was attributed to the variation within populations (Table 6). Similarly the G_st_ (0.32) value also confirmed high genetic differentiation among populations. The higher genetic differentiation might be due to genetic drift resulted from small population size and limited gene flow. AMOVA analysis based on further grouping of the populations into regions and zones resulted in non-significant variation. The absence of genetic divergence between regions and among zones could be attributed to the widespread practice of exchanging seeds and planting material between relatives and migrants from one location to the other during trade exchange.

Gene flow through a movement of individuals on a geographic scale [20], in conjunction with other evolutionary forces, can result in the spread of single genes (or DNA sequences). A value of N_m_ smaller than 2 provides a substantial opportunity for genetic population divergence [34]. The level of gene flow (N_m_) was estimated to be 1.08 individuals per generation between populations (Table 4), suggesting that gene exchange between populations was limited but with some degree of human mediated genetic material transfer. This result is in accordance with the AMOVA result and G_st_ (0.32) value which shows high variation among populations.

### Genetic distance and cluster analysis

In this study, the inter-population genetic distance ranged from 0.06 to 0.41 (Table 5). The maximum genetic distance was observed between Jimma II and Bonga III populations. This might be due to the high geographic distance between the two populations. Bonga I and Bonga III populations found to be more related with each other showing low genetic distance (0.06). The similarity between these two populations may be due to their occurrence in the same zone and the use of homogeneous korarima landraces in the various household farms in the two populations. Both NJ tree (Fig. 3) and the UPGMA dendrogram (Fig. 4) also revealed these two populations to be closely related as they are found in the same cluster. Farm based biodiversity study conducted by Eyob et al. [2] also confirmed the same result in a way that similarities between pairs of sites in Keffa zone measured by Sørenson’s similarity Index found to be uniform, indicating homogenous korarima landraces in the various household farms in Keffa zone [2].

The present study did not reveal a clear pattern of clustering of populations according to their zone of origin. UPGMA tree topology allowed the identification of two major groups (cluster I and II) (Fig. 4). The first cluster formed three sub clusters from which one of them constituted populations from Bench magi zone without mixing with any other population. This can support the high degree of genetic differentiation (G_st_ = 0.32) obtained in this study. No clear grouping of individuals from the same population is observed in the NJ dendrogram (Fig. 3) where in the three clusters, individuals from one population were clustered with individuals from different populations. These results also became apparent from both the 2D (Fig. 5) and 3D (Fig. 6) PCoA analysis in this study. The failure of most korarima populations and individuals to cluster on the basis of their respective zone and populations, respectively, could be due to the presence of gene flow between and among local populations and between adjacent zones mainly in the form of human mediated transfer of genetic material. This may be due to the close geographical proximity of sampled plants (Fig. 1) contributing to their genetic similarity. In addition, the common result revealed from NJ and UPGMA cluster showed populations from geographically distant locations, Jimma and Tepi, to be clustered together. This indicates that populations from the two locations are genetically closely related because they are either originated from the same ancestor or could be due to exchange of genetic material between the two localities.

## Conclusions

In the present study, seven ISSR markers have been used to study the genetic variability of *A. corrorima* populations from southwestern Ethiopia. The results obtained, validate once more that ISSR are useful markers in genetic diversity studies, due to the very high level of polymorphism detected by the primers. The findings should be taken into account when conservation actions, management policies for the species and site identification for in situ and ex situ conservation strategies are developed. Among the thirteen populations, all genetic diversity estimators indicate higher genetic diversity in Mizan Teferi II population. Therefore, this population should be considered as a key site in designing conservation strategies for this crop. Populations like Jimma I and Jimma II showed the lowest genetic diversity and should also be considered during conservation strategies due to the putative risk of extinction that they face because of low genetic diversity.

## Abbreviations

AMOVA: analysis of molecular variance
GD: genetic diversity
SSR: inter simple sequence repeats
NJ: neighbor joining
NPB: number of polymorphic bands
PCoA: principal coordinate analysis
PCR: polymerase chain reaction
PPB: percentage polymorphic bands
RAPD: random amplified polymorphic DNA
RFLP: restriction fragment length polymorphism
SNNP: Southern Nations, Nationalities, and Peoples’ Region
SW: southwestern
UPGMA: unweighted pair group method with arithmetic mean
